# Prediagnosis Insights Into Amyotrophic Lateral Sclerosis: Clinical Symptoms and Medication Use

**DOI:** 10.1002/jcsm.70003

**Published:** 2025-07-11

**Authors:** Chunyang Pang, Wen Cao, Jiali Xie, Yaojia Li, Luyi Zhu, Huan Yu, Dongsheng Fan, Binbin Deng

**Affiliations:** ^1^ Department of Rehabilitation Medicine First Affiliated Hospital of Wenzhou Medical University Wenzhou China; ^2^ Department of Neurology First Affiliated Hospital of Wenzhou Medical University Wenzhou China; ^3^ Department of Neurology Peking University Third Hospital Beijing China; ^4^ Department of Neurology, Shanghai East Hospital, School of Medicine Tongji University Shanghai China; ^5^ Department of Pediatrics Second Affiliated Hospital and Yuying Children's Hospital of Wenzhou Medical University Wenzhou China

**Keywords:** amyotrophic lateral sclerosis, medications, prodromal symptoms

## Abstract

**Background:**

Amyotrophic lateral sclerosis (ALS) has a prolonged latency period, though its preclinical characteristics remain poorly understood. This study uses UK Biobank data to explore and compare ALS's pre‐diagnostic features, including symptoms and medication use, aiming to provide insights into the disease's underlying mechanisms.

**Methods:**

Clinical symptoms and medications were identified from self‐reports, hospital records, and death registry data. Propensity score matching was used to match ALS with Alzheimer's disease (ad) and Parkinson's disease (PD), ensuring balance in socioeconomic factors to compare symptoms 0–5 years before diagnosis. Cox regression analysis was applied to assess the associations between medication use and the risk of incident ALS and mortality after ALS diagnosis.

**Results:**

A total of 753 ALS cases were observed in 502 417 participants, with an incidence rate of 10.58 per 100 000 person‐years. In the ALS cohort, the male‐to‐female ratio was 2.9, with a median age at onset of 64.61 years (Interquartile range (IQR): 56.80–71.31) and a median survival time post‐diagnosis of 9.08 months (IQR: 3.18–18.98), while females (log‐rank *p* = 0.038) and individuals with earlier (< 64.61 years) disease onset (log‐rank *p* < 0.001) had longer survival periods. In the 5 years prior to diagnosis, ALS showed a higher incidence of falls compared to ad (11.3% vs. 3.2%, *p* < 0.001), but a lower incidence than PD (10.7% vs. 28.3%, *p* < 0.001). Additionally, ALS had a lower incidence of depression (4.6% vs. 25.6%, *p* < 0.001), anxiety (3.5% vs. 18.1%, *p* < 0.001), sleep disorders (1.4% vs. 7.2%, *p* < 0.001), hypotension (3.4% vs. 30.5%, *p* < 0.001), constipation (0.3% vs. 4.9%, *p* < 0.001), and urinary dysfunction (2.2% vs. 8.7%, *p* < 0.001) compared with PD. The use of calcium channel blockers may be a risk factor for incident ALS (adjusted HR 1.61, 95% CI: 1.22–2.12, *p* < 0.001).

**Conclusions:**

Pre‐diagnostic presentations of falls are more frequent in ALS than in ad, but less frequent than in PD. However, ALS exhibits fewer psychiatric symptoms and autonomic dysfunction compared with PD. The use of calcium channel blockers may be associated with an increased risk of developing ALS in the future.

## 1 Introduction

Amyotrophic lateral sclerosis (ALS) is characterized by the progressive degeneration of neurons responsible for controlling voluntary muscle movement [1]. Due to the lack of effective therapeutic interventions, mortality often occurs within 3 years of the onset of symptoms, primarily due to respiratory failure [2]. Although genetics is a major risk factor for ALS [3], it does not fully account for the entire disease burden, as the causes of 85% of sporadic cases remain unclear [4]. A gene‐time‐environment hypothesis of ALS posits that the disease arises from an interplay of genetic predisposition, age‐associated cellular damage, and environmental exposure [5]. However, the complex interactions of multiple factors contributing to ALS and its heterogeneous manifestations make research on non‐genetic factors in ALS challenging [6]. On the other hand, early diagnosis of ALS remains a significant challenge. While ALS is still a relatively rare disease, its prevalence is expected to rise with ageing populations and improved diagnosis methods [7], maintaining a global incidence rate of only 1.68 per 100 000 person‐years [8]. Additionally, research on ALS‐specific early symptoms that can be easily differentiated from those of other neurodegenerative diseases is still insufficient, leading to delayed or missed diagnoses [9].

Research on the preclinical features of the disease may help provide some evidence. As a neurodegenerative disease, ALS is considered to have a prolonged preclinical phase, similar to disorders such as Parkinson's disease (PD), where symptoms can emerge 15 years earlier [10], and Alzheimer's disease (ad), marked by amyloid plaque build‐up up to 20 years before diagnosis [11]. Thus, it is reasonable to speculate that there may be specific pre‐disease features associated with ALS. Prior ALS research predominantly concentrates on the characteristics and treatments of diagnosed ALS patients, mainly through case–control or cross‐sectional studies. There is a lack of research on the preclinical stage of the disease, with limited evidence available, especially for sporadic cases, which constitute the majority of ALS cases [12].

Against this backdrop, we utilized the data from the UK Biobank's registry of over 500 000 individuals to conduct further research on ALS. The UK Biobank is a large‐scale biomedical database and research resource, containing in‐depth genetic and health information from half a million UK participants [13]. Using this valuable resource, our study aimed to explore: (1) the incidence, sociodemographic characteristics, and prognosis of ALS; (2) preclinical features of ALS and how they compare with those of individuals who later develop PD and ad; and (3) prospective analyses identifying clinical medications linked to ALS risk and its progression.

## 2 Method

### 2.1 Study Population and ALS Diagnosis

Among the 502 357 participants recruited by the UK Biobank, aged 37–73, from 22 assessment centres across England, Wales, and Scotland between 2006 and 2010 [14], a total of 753 were diagnosed with ALS. Additionally, the study included 55 292 healthy controls, 4809 individuals with PD, and 4445 individuals with ad. Healthy controls were defined based on the absence of International Classification of Diseases (ICD)‐coded diseases. Clinical outcomes were determined over a follow‐up period from 2007 to 2023 using algorithmically defined outcomes (ALS [field ID 42029], ad [field ID 42020], PD [field ID 42032]), derived from encoded data collected during the UK Biobank's initial assessment, hospital admission records, and mortality data, with the algorithm's accuracy validated in a study of 17 000 Biobank participants in England and used in several previous studies [15, 16]. This research was conducted using the UK Biobank Resource under Application Number 108832 and informed consent was obtained from all participants registered in the UK Biobank (https://www.ukbiobank.ac.uk/learn‐more‐about‐uk‐biobank/about‐us/ethics).

### 2.2 Study Design

**FIGURE 1:**
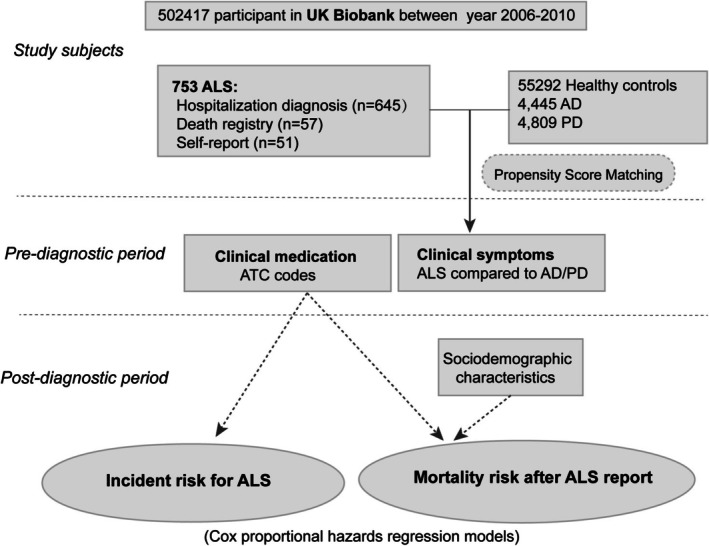
Research design flowchart. AD, Alzheimer's disease; ALS, amyotrophic lateral sclerosis; ATC, Anatomical Therapeutic Chemical Classification System; BMI, body mass index; PD, Parkinson's disease; TDI, Townsend Deprivation Index.

As depicted in Figure 1, our study focuses on ALS and delves into its sociodemographic characteristics, clinical symptoms, and medications. We comprehensively analysed 753 ALS patients, detailing their age, gender, Townsend Deprivation Index (TDI), ethnicity, and education, among other aspects. In our exploration of ALS disease risk, we maintained focus on these 753 patients. For the post‐diagnosis survival analysis, we excluded patients reported only in death registries (*n* = 57) and those who self‐reported ALS (*n* = 51), due to potential inaccuracies in diagnosis timing. Patients without a recorded death event by the end of follow‐up were also excluded (*n* = 139), as their survival time remained indeterminate. Consequently, 506 patients were included in the descriptive analysis and Cox regression for survival post‐diagnosis.

### 2.3 Clinical Symptoms

The clinical features associated with ad and PD, as used in prior research [17], were thoroughly considered in the study. These include memory problems, sleep disorders, constipation, anxiety, depression, falls, hypotension, urinary dysfunction, and abnormal weight loss. All events were identified from baseline interviews and updated according to ICD‐10 coding up until the end of the follow‐up period (Table S1). The timing of the first occurrence of these events was determined using the corresponding dates in ICD‐10 codes or from the dates when questionnaires were conducted. The clinical features comparisons across ALS, PD, and ad were conducted among matched cohorts after propensity score matching. Matching factors included sex, age, qualifications, ethnicity, and TDI with a calliper value of 0.2 times the standard deviation of propensity scores.

### 2.4 Medications and Covariates

Medications were self‐reported (Field ID: 20003) and classified based on the Anatomical Therapeutic Chemical (ATC) classification system [18], encompassing all categories of antihypertensive drugs, laxatives, statins, and medications used to treat neurological disorders, including treatments for depression and anxiety conditions (Table S2). Additionally, we further classified the pharmacological mechanisms of the aforementioned drug categories, with the detailed classification listed in Table S3.

Covariates, including age, sex, ethnicity, qualifications, and TDI, were obtained during the initial assessment. Educational attainment was self‐reported and categorized into academic (college or university degree, A‐levels or equivalent, general certificate of secondary education general certificate of secondary education [GCSEs] or equivalent) and vocational (certificate of secondary education [CSEs] or equivalent, national vocational qualification [NVQ] or higher national diploma [HND] or higher national certificate [HNC] or equivalent, other vocational qualifications) education. The TDI was derived from national census data, including information on unemployment, vehicle ownership, household overcrowding, and occupation.

### 2.5 Statistical Analysis

Baseline and clinical characteristics were summarized as median (Interquartile range (IQR)) for continuous variables and as counts (percentages) for categorical variables. In the analysis of prodromal features, the cumulative incidence of each health condition across different time windows before and after ALS onset was used to demonstrate their progression over time. Propensity scores were generated based on covariates (sex, age, qualifications, ethnicity, and TDI) and matched using nearest‐neighbour matching with a calliper of 0.2 times the standard deviation to compare the occurrence rates of nine conditions among patients with different diseases. Additionally, the Chi‐squared test was used to compare categorical variables, particularly the occurrence rates of health conditions, in samples with *n* ≥ 40 and a theoretical frequency (T) ≥ 5. Associations between medications and ALS risk and prognosis were analysed using Cox proportional hazard models. In the time‐to‐death analysis, Kaplan–Meier curves were used to illustrate survival differences among various groups. The follow‐up period commenced from the date of the initial ALS diagnosis until the date of death. The proportional hazards assumptions were examined using Schoenfeld residuals. All analyses were adjusted for sex, age at admission, qualifications, ethnicity, and TDI. Missing covariate data (missing percentage: TDI‐0.12%, Qualifications‐19.00%) were imputed using the multiple imputation method with the ‘mice’ package in R.

All statistical analyses were performed with R software, version 4.2.1. Two‐sided *p* < 0.05 was considered statistically significant. Bonferroni correction was applied for multiple comparisons.

## 3 Results

### 3.1 Baseline Characteristics

The initial UK Biobank cohort, consisting of 502 417 participants, featured a diverse ethnic composition: 94.6% were White, 1.6% Black, 2.0% South Asian, with the remainder from other mixed backgrounds. The incidence of ALS in this cohort was 10.58 per 100 000 person‐years of follow‐up: 10.56 in the White cohort, 17.59 in the Black cohort, and 7.14 in the South Asian cohort. Table 1 presents the sociodemographic characteristics of 753 ALS patients. Of these, 645 were diagnosed through hospital records, 57 were identified via death registries, and 51 were confirmed through self‐reports. In the ALS cohort, which was predominantly white (93.9%), the patients had a median admission age of 57 years (IQR: 50–63, Birth year: 1937–1969). A significant majority (77.7%) held vocational qualifications, and the cohort displayed a wide range of socio‐economic statuses, as reflected by the median TDI score (−2.17, IQR: −3.68 to 0.36). Furthermore, a notable male predominance was evident in the cohort, with a male‐to‐female ratio of 2.9, and among those diagnosed in hospitals, the median age at onset was 64.61 years (IQR: 56.80–71.31). Additionally, compared with participants without ALS, the ALS cohort showed a higher male‐to‐female ratio, a greater proportion of individuals with vocational qualifications, and an older average age, as elaborated in Table S4.

**TABLE 1:** Baseline characteristics of all included patients with ALS.

	Overall ALS (*n* = 753)	Hospitalization (*n* = 645)	Death (*n* = 57)	Self‐report (*n* = 51)	*p* value
Sex, *n* (%)					
Female	425 (56.4)	366 (56.7)	32 (56.1)	27 (52.9)	
Male	328 (43.6)	279 (43.3)	25 (43.9)	24 (47.1)	0.869
Male to female ratio	1.30	1.31	1.28	1.13	
Year of birth, *n* (%)					0.027
1937–1940	95 (12.6)	77 (11.9)	13 (22.8)	5 (9.8)	
1940–1950	457 (60.7)	397 (61.6)	34 (59.6)	26 (51.0)	
1959–1960	164 (21.8)	136 (21.1)	10 (17.5)	18 (35.3)	
1960–1969	37 (4.9)	35 (5.4)	0 (0.0)	2 (3.9)	
Age at admission	57.00 (50.00–63.00)	57.00 (50.00–63.00)	56.00 (48.00–63.00)	60.00 (51.50–63.00)	0.668
TDI	−2.17 (−3.68–0.36)	−2.25 (−3.73–0.28)	−1.95 (−3.67–0.35)	−1.74 (−3.27–1.45)	0.388
Qualification, *n* (%)					0.454
Academic	496 (77.7)	432 (78.0)	32 (71.1)	32 (82.1)	
Vocational	142 (22.3)	122 (22.0)	13 (28.9)	7 (17.9)	
Ethnicity, *n* (%)					0.673
White	707 (93.9)	605 (93.8)	55 (96.5)	47 (92.2)	
Black	20 (2.7)	16 (2.5)	1 (1.8)	3 (5.9)	
South Asian	10 (1.3)	10 (1.6)	0 (0.0)	0 (0.0)	
Other mix	16 (2.1)	14 (2.2)	1 (1.8)	1 (2.0)	
Age at ALS report, years	63.92 (56.09–70.67)	64.61 (56.80–71.31)	66.21 (59.30–72.47)	52.42 (42.77–57.40)	< 0.001
Death, *n* (%)	576 (76.5)	506 (78.4)	57 (100.0)	13 (25.5)	< 0.001
Age at death	71.40 (65.70–75.60)	71.10 (65.50–75.50)	73.10 (68.10–76.40)	66.50 (61.20–68.40)	0.004
Survival time, months	7.75 (1.63–18.29)	9.08 (3.18–18.98)	0.00 (0.00–0.00)	43.13 (30.47–72.43)	<0.001

Abbreviations: ALS, amyotrophic lateral sclerosis; TDI, Townsend Deprivation Index.

In the survival analysis, after excluding ALS patients diagnosed outside of hospitals and those with unknown survival times, a total of 506 ALS patients were included in the analysis. As shown in Figure S2, the median survival time post‐diagnosis was 9.08 months (IQR: 3.18–18.98). Regarding survival advantages, female (log‐rank *p* = 0.038) and those with earlier onset of the disease had longer survival periods (log‐rank *p* < 0.001). However, no significant differences in survival time were observed across different ages, ethnicities, or TDI scores.

### 3.2 Clinical Symptoms

**FIGURE 2:**
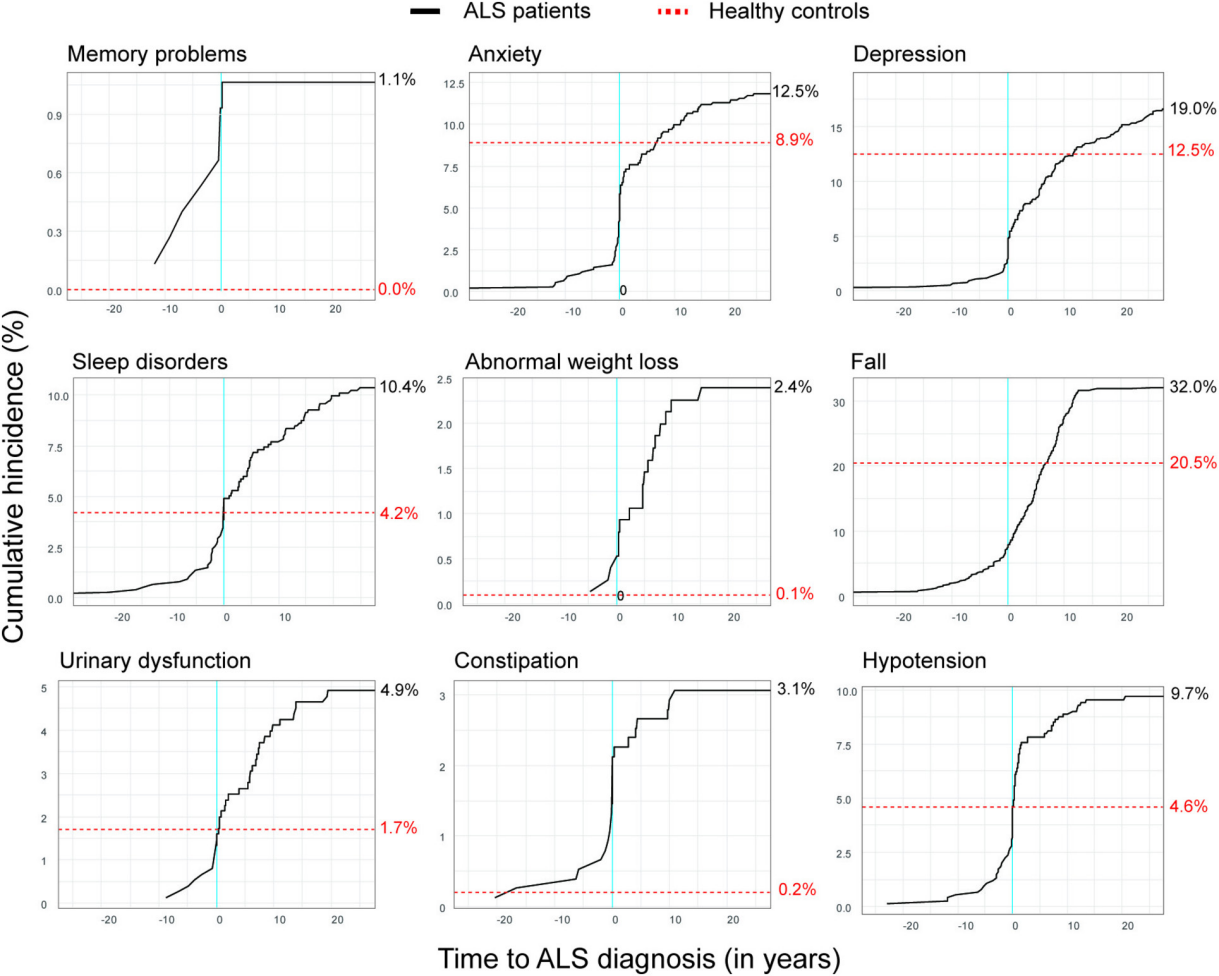
Cumulative timeline summary of multiple clinical symptoms in patients with ALS. Abbreviations: ALS, amyotrophic lateral sclerosis.

The cumulative incidence of various symptoms across different time windows surrounding ALS report is illustrated in a Figure 2: starting 10 years prior to ALS diagnosis, the frequency of symptoms (memory problems, sleep disorders, constipation, anxiety, depression, falls, hypotension, urinary dysfunction, and abnormal weight loss) begins to rise, with a more rapid increase observed around the time of diagnosis. The prevalence of most symptoms in ALS patients was higher than that in healthy controls, including memory problems (1.1% vs. 0.0%, *p* = 0.023), depression (19.0% vs. 12.5%, *p* = 0.002), sleep disorders (10.4% vs. 4.2%, *p* < 0.001), abnormal weight loss (2.4% vs. 0.1%, *p* < 0.001), falls (32.0% vs. 20.0%, *p* < 0.001), urinary dysfunction (4.9% vs. 1.7%, *p* = 0.002), and constipation (3.1% vs. 0.2%, *p* < 0.001). However, no statistically significant difference was found in hypotension (9.7% vs. 4.6%, *p* = 0.058) and anxiety (12.5% vs. 8.9%, *p* = 0.054).

Among nearest‐neighbour matching, coarsened exact matching (CEM), exact matching, and optimal matching, the nearest‐neighbour matching model was selected as the optimal approach based on standardized mean differences (SMD < 0.1) and sample size (Table S5). Using propensity score matching (PSM) based on covariates (sex, age, qualifications, ethnicity, and TDI), 441 ad patients and 597 PD patients were matched 1:1 with ALS patients. Additionally, using PSM based on covariates (sex, age, imputed qualifications, ethnicity, and imputed TDI), 560 ad patients and 716 PD patients were matched 1:1 with ALS patients. A detailed summary of the matching balance is summarized in Table S5 and Figure S1.

**FIGURE 3:**
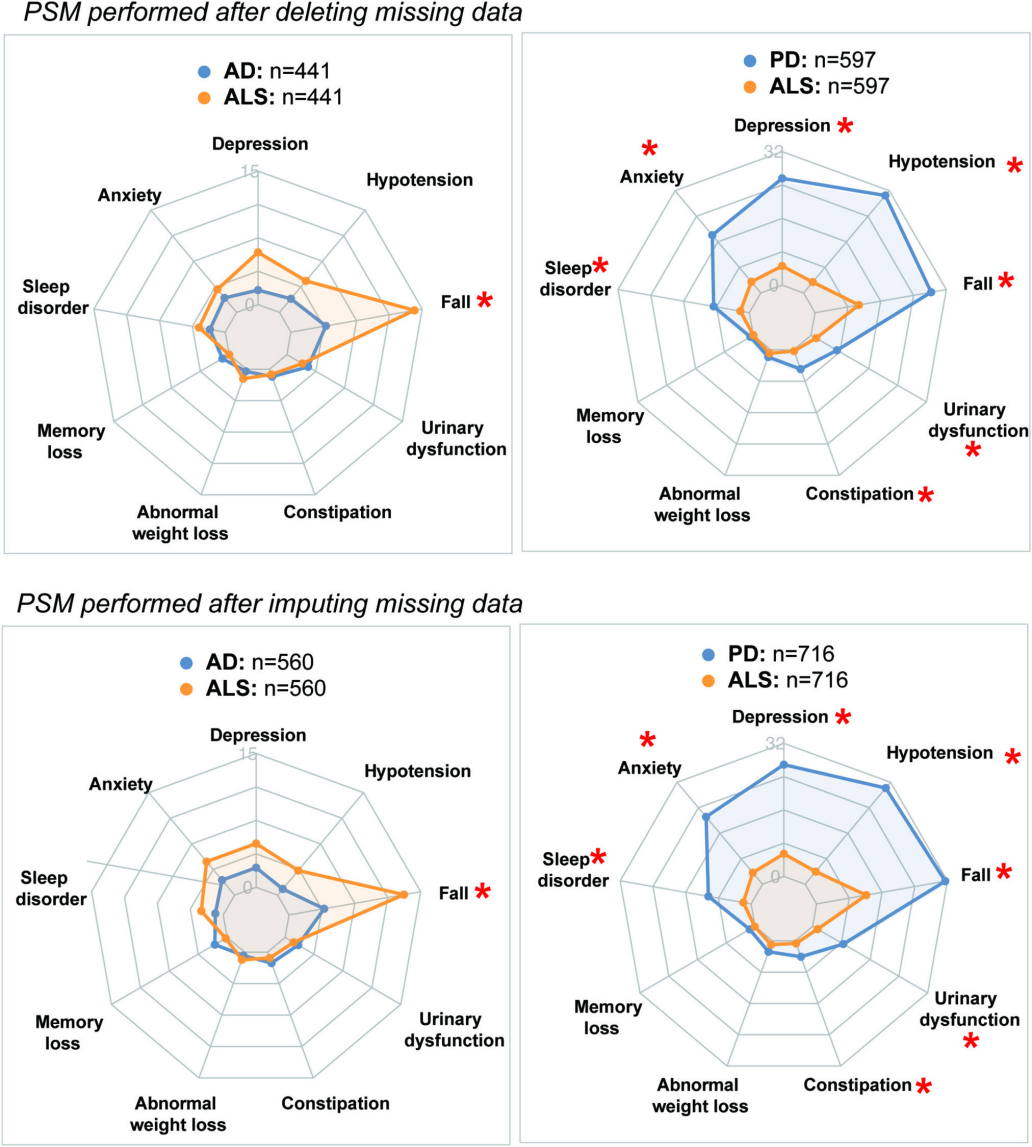
Prevalence of health conditions in ALS cohorts compared with those with ad and PD within the 0–5 years preceding diagnosis. *Differences were considered significant after Bonferroni correction (*p* < 0.0056). Abbreviations: AD, Alzheimer's disease; ALS, amyotrophic lateral sclerosis; PD, Parkinson's disease; PSM, propensity score matching.

As shown in Figure 3 and Table 2, in the 0–5 years preceding diagnosis, falls were more common in ALS patients compared with those with ad (11.3% vs. 3.2%, *p* < 0.001). However, compared with PD, ALS patients had a lower prevalence of falls (10.7% vs. 28.3%, *p* < 0.001), depression (4.6% vs. 25.6%, *p* < 0.001), anxiety (3.5% vs. 18.1%, *p* < 0.001), hypotension (3.4% vs. 30.5%, *p* < 0.001), constipation (0.3% vs. 4.9%, *p* < 0.001), urinary dysfunction (2.2% vs. 8.7%, *p* < 0.001), and sleep disorders (1.4% vs. 7.2%, *p* < 0.001). PSM performed using sex, age, imputed qualifications, ethnicity, and imputed TDI yielded consistent results.

**TABLE 2:** Prevalence of clinical symptoms in ALS cohorts compared with ad and PD within the 0–5 years preceding diagnosis.

	Vs. ad			Vs. PD		
	Controls	ALS	*p* value	Controls	ALS	*p* value
**PSM performed after deleting missing data**						
*n*	441	441		597	597	
Fall	12 (3.2)	39 (11.3)	< 0.001	169 (28.3)	49 (10.7)	< 0.001
Depression	5 (1.3)	18 (4.7)	0.010	153 (25.6)	23 (4.6)	< 0.001
Anxiety	7 (1.7)	11 (2.7)	0.455	108 (18.1)	19 (3.5)	< 0.001
Memory loss	3 (0.7)	0 (0.0)	0.250	5 (0.8)	0 (0.0)	0.075
Hypotension	7 (1.6)	15 (3.7)	0.107	182 (30.5)	19 (3.4)	< 0.001
Abnormal weight loss	1 (0.2)	4 (0.9)	0.371	11 (1.8)	5 (0.9)	0.219
Constipation	3 (0.7)	2 (0.5)	0.977	29 (4.9)	2 (0.3)	< 0.001
Urinary dysfunction	6 (1.4)	10 (2.4)	0.404	52 (8.7)	12 (2.2)	< 0.001
Sleep disorders	9 (2.2)	7 (1.6)	0.760	43 (7.2)	8 (1.4)	< 0.001
**PSM performed after imputing missing data**						
*n*	560	560		716	716	
Fall	19 (4.0)	56 (13.1)	< 0.001	192 (29.5)	63 (11.4)	< 0.001
Depression	11 (2.2)	24 (4.9)	0.032	164 (25.2)	31 (5.1)	< 0.001
Anxiety	11 (2.2)	24 (4.9)	0.032	129 (19.8)	22 (3.4)	< 0.001
Memory loss	9 (1.6)	1 (0.2)	0.026	9 (1.4)	1 (0.1)	0.018
Hypotension	5 (0.9)	19 (3.6)	0.006	184 (28.3)	25 (3.7)	< 0.001
Abnormal weight loss	2 (0.4)	5 (0.9)	0.450	17 (2.6)	6 (0.9)	0.022
Constipation	7 (1.3)	4 (0.7)	0.508	25 (3.8)	4 (0.6)	< 0.001
Urinary dysfunction	5 (0.9)	13 (2.5)	0.079	64 (9.8)	12 (1.8)	< 0.001
Sleep disorders	9 (1.7)	6 (1.1)	0.595	52 (8.0)	9 (1.3)	< 0.001

*Note:* Participants were matched using PSM based on sex, age, qualifications, ethnicity, and TDI.

Abbreviations: ad, Alzheimer's disease; ALS, amyotrophic lateral sclerosis; PD, Parkinson's disease; PSM, propensity score matching.

Additionally, we conducted a symptom comparison in ALS patients, categorizing them by the timing of disease onset, length of survival period, and sex. We found that early‐onset ALS patients exhibited higher rates of depression (22.0% vs. 16.0%, *p* = 0.043) and falls (35.5% vs. 28.5%, *p* = 0.045) compared with those with late‐onset ALS. Furthermore, patients with shorter survival periods were more likely to experience anxiety than those with longer survival periods (15.6% vs. 9.0%, *p* = 0.023) (Table S6).

### 3.3 Medication

**FIGURE 4:**
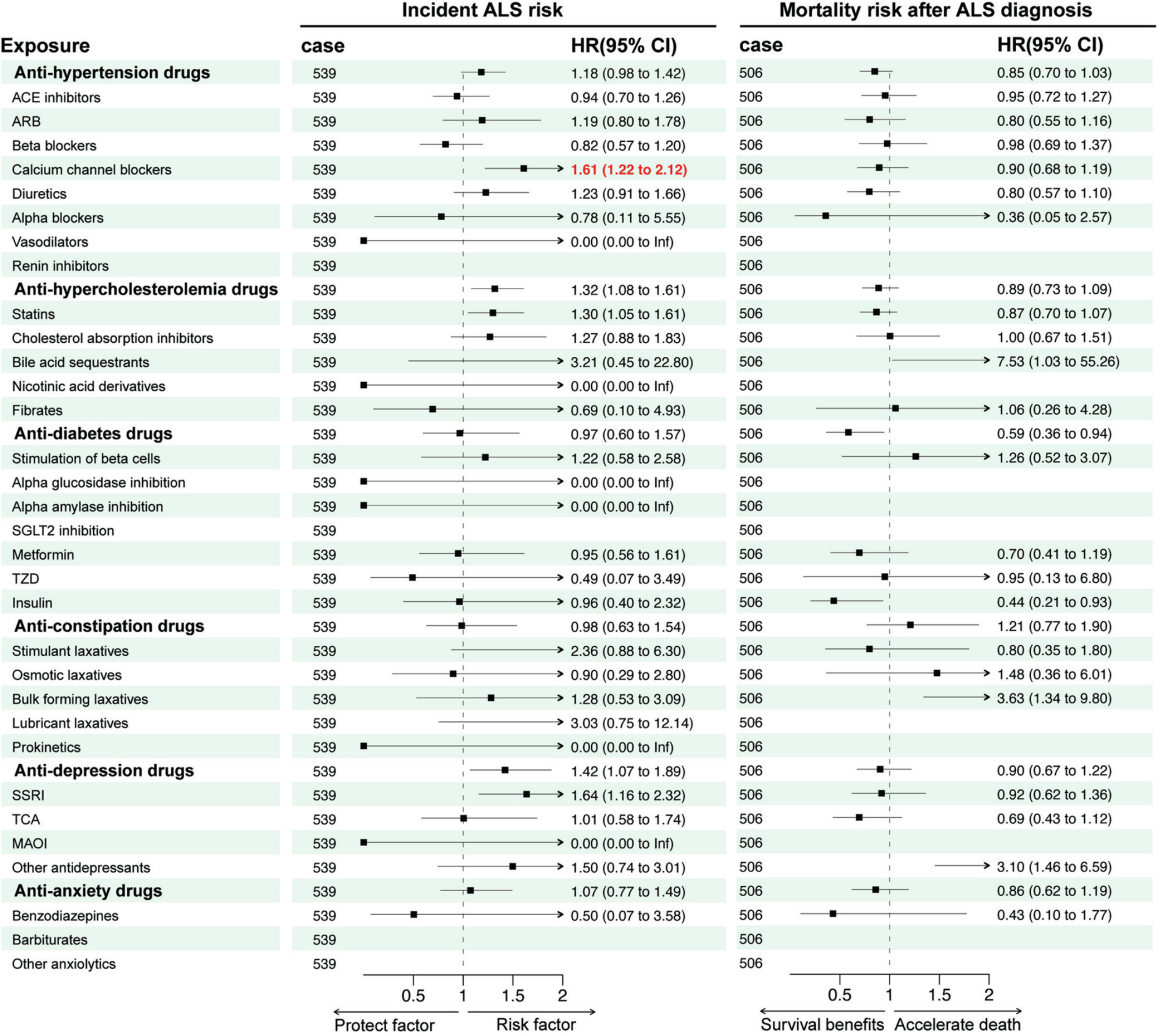
Associations between medication use and the risk of incident ALS and mortality after ALS diagnosis. The Cox regression analysis was adjusted for sex, age, qualifications, ethnicity, TDI, and BMI. Red highlights indicate statistical significance after Bonferroni correction (*p* < 0.0013). Abbreviations: ACE, angiotensin‐converting enzyme; ALS, amyotrophic lateral sclerosis; ARB, angiotensin II receptor blocker; BMI, body mass index; HR, hazard ratio; MAOI, monoamine oxidase inhibitor; SGLT2, sodium‐glucose cotransporter‐2; SSRI, selective serotonin reuptake inhibitor; TCA, tricyclic antidepressant; TDI, Townsend Deprivation Index; TDZ, thiazolidinedione.

As shown in Figure 4 and Table 3, calcium channel blockers (HR (95% CI) = 1.69 (1.31–2.17), *p* < 0.001), antihypercholesterolaemia drugs (HR (95% CI) = 1.36 (1.14–1.64), *p* = 0.001), and statins (HR (95% CI) = 1.39 (1.15–1.69), *p* = 0.001) may be risk factors for incident ALS. After adjusting for sex, age, education level, ethnicity, TDI, and body mass index (BMI), calcium channel blockers remained significant (HR (95% CI) = 1.61 (1.22–2.12), *p* = 0.001).

**TABLE 3:** Medications use and incident ALS risk, excluding patients with follow‐up less than 2 years.

	Unadjusted			Adjusted		
Index	ALS case/n	HR (95% CI)	*p* value	ALS case/n	HR (95% CI)	*p* value
**Antihypertension drugs**	630/55530	1.27 (1.07–1.51)	0.005	539/49448	1.18 (0.98–1.42)	0.078
ACE inhibitors	630/55530	1.03 (0.79–1.34)	0.815	539/49448	0.94 (0.70–1.26)	0.674
ARB	630/55530	1.50 (1.08–2.09)	0.017	539/49448	1.19 (0.80–1.78)	0.393
Beta blockers	630/55530	0.98 (0.71–1.35)	0.897	539/49448	0.82 (0.57–1.20)	0.305
Calcium channel blockers	630/55530	1.69 (1.31–2.17)	< 0.001*	539/49448	1.61 (1.22–2.12)	0.001*
Diuretics	630/55530	1.34 (1.02–1.75)	0.034	539/49448	1.23 (0.91–1.66)	0.185
Alpha blockers	630/55530	0.71 (0.10–5.08)	0.737	539/49448	0.78 (0.11–5.55)	0.805
Vasodilators	630/55530	0 (0‐Inf)	0.989	539/49448	0 (0‐Inf)	0.990
Renin inhibitors	630/55530	—	—	539/49448	—	—
**Antihypercholesterolaemia drugs**	630/55530	1.36 (1.14–1.64)	0.001*	539/49448	1.32 (1.08–1.61)	0.006
Statins	630/55530	1.39 (1.15–1.69)	0.001*	539/49448	1.30 (1.05–1.61)	0.017
Cholesterol absorption inhibitors	630/55530	1.15 (0.81–1.64)	0.435	539/49448	1.27 (0.88–1.83)	0.204
Bile acid sequestrants	630/55530	2.80 (0.39–19.92)	0.303	539/49448	3.21 (0.45–22.80)	0.245
Nicotinic nacid derivatives	630/55530	0 (0‐Inf)	0.985	539/49448	0 (0‐Inf)	0.986
Fibrates	630/55530	0.61 (0.09–4.32)	0.619	539/49448	0.69 (0.10–4.93)	0.714
**Antidiabetes drugs**	630/55530	0.98 (0.63–1.52)	0.918	539/49448	0.97 (0.60–1.57)	0.887
Stimulation of beta cells	630/55530	1.04 (0.49–2.20)	0.914	539/49448	1.22 (0.58–2.58)	0.598
Alpha glucosidase inhibition	630/55530	0 (0‐Inf)	0.989	539/49448	0 (0‐Inf)	0.989
Alpha amylase inhibition	630/55530	0 (0‐Inf)	0.989	539/49448	0 (0‐Inf)	0.989
SGLT2 inhibition	630/55530	—	—	539/49448	—	—
Metformin	630/55530	0.93 (0.56–1.52)	0.765	539/49448	0.95 (0.56–1.61)	0.848
TZD	630/55530	0.40 (0.06–2.87)	0.365	539/49448	0.49 (0.07–3.49)	0.476
Insulin	630/55530	1.36 (0.68–2.73)	0.387	539/49448	0.96 (0.40–2.32)	0.933
**Anticonstipation drugs**	630/55530	1.26 (0.87–1.83)	0.223	539/49448	0.98 (0.63–1.54)	0.946
Stimulant laxatives	630/55530	3.00 (1.34–6.70)	0.007	539/49448	2.36 (0.88–6.30)	0.088
Osmotic laxatives	630/55530	1.63 (0.73–3.65)	0.231	539/49448	0.90 (0.29–2.80)	0.854
Bulk forming laxatives	630/55530	1.38 (0.62–3.07)	0.437	539/49448	1.28 (0.53–3.09)	0.582
Lubricant laxatives	630/55530	2.73 (0.68–10.96)	0.155	539/49448	3.03 (0.75–12.14)	0.118
Prokinetics	630/55530	0 (0‐Inf)	0.989	539/49448	0 (0‐Inf)	0.990
**Antidepression drugs**	630/55530	1.44 (1.11–1.86)	0.006	539/49448	1.42 (1.07–1.89)	0.015
SSRI	630/55530	1.46 (1.05–2.05)	0.026	539/49448	1.64 (1.16–2.32)	0.005
TCA	630/55530	1.20 (0.75–1.91)	0.451	539/49448	1.01 (0.58–1.74)	0.986
MAOI	630/55530	0 (0‐Inf)	0.989	539/49448	0 (0‐Inf)	0.989
Other antidepressants	630/55530	1.73 (0.95–3.14)	0.071	539/49448	1.50 (0.74–3.01)	0.257
**Antianxiety drugs**	630/55530	1.15 (0.86–1.55)	0.343	539/49448	1.07 (0.77–1.49)	0.674
Benzodiazepines	630/55530	0.45 (0.06–3.18)	0.422	539/49448	0.50 (0.07–3.58)	0.492
Barbiturates	630/55530	—	—	539/49448	—	—
Other anxiolytics	630/55530	—	—	539/49448	—	—

*Note:* The Cox regression analysis was adjusted for sex, age, qualifications, ethnicity, TDI, and BMI. Bold formatting has been applied exclusively to medication categories to denote classifications.

Abbreviations: ACE, angiotensin‐converting enzyme; ALS, amyotrophic lateral sclerosis; ARB, angiotensin II receptor blocker; BMI, body mass index; HR, hazard ratio; MAOI, monoamine oxidase inhibitor; SGLT2, sodium‐glucose cotransporter‐2; SSRI, selective serotonin reuptake inhibitor; TCA, tricyclic antidepressant; TDI, Townsend Deprivation Index; TDZ, thiazolidinedione.

* Significance after Bonferroni corrections, with a corrected threshold of *p* < 0.0013.

The Cox regression results of medication use and mortality risk after ALS diagnosis are summarized in Figure 4 and Table 4. In the unadjusted model, antidiabetic drugs [HR (95% CI) = 0.57 (0.35–0.91), *p* = 0.018] and insulin [HR (95% CI) = 0.44 (0.21–0.94), *p* = 0.034] may be protective factors for prognosis, while bulk‐forming laxatives [HR (95% CI) = 3.49 (1.30–9.38), *p* = 0.013] and other antidepressants [HR (95% CI) = 2.78 (1.32–5.89), *p* = 0.007] may be risk factors. After adjusting for sex, age, education level, ethnicity, TDI, and BMI, antidiabetic drugs [HR (95% CI) = 0.59 (0.36–0.94), *p* = 0.028] and insulin [HR (95% CI) = 0.44 (0.21–0.93), *p* = 0.033] remained protective, while bulk‐forming laxatives [HR (95% CI) = 3.63 (1.34–9.80), *p* = 0.011] and other antidepressants [HR (95% CI) = 3.10 (1.46–6.59), *p* = 0.003] remained risk factors. However, after Bonferroni multiple correction, all of these associations lost significance.

**TABLE 4:** Medications use and incident mortality risk after ALS diagnosis.

	Unadjusted			Adjusted		
Index	ALS case/n	HR (95% CI)	*p* value	ALS case/n	HR (95% CI)	*p* value
**Antihypertension drugs**	506/506	0.88 (0.73–1.06)	0.186	506/506	0.85 (0.70–1.03)	0.095
ACE inhibitors	506/506	0.98 (0.74–1.30)	0.885	506/506	0.95 (0.72–1.27)	0.750
ARB	506/506	0.83 (0.57–1.20)	0.314	506/506	0.80 (0.55–1.16)	0.238
Beta blockers	506/506	0.95 (0.68–1.34)	0.777	506/506	0.98 (0.69–1.37)	0.886
Calcium channel blockers	506/506	0.95 (0.72–1.25)	0.694	506/506	0.90 (0.68–1.19)	0.443
Diuretics	506/506	0.84 (0.61–1.15)	0.274	506/506	0.80 (0.57–1.10)	0.169
Alpha blockers	506/506	0.31 (0.04–2.24)	0.247	506/506	0.36 (0.05–2.57)	0.308
Vasodilators	506/506	—	—	506/506	—	—
Renin inhibitors	506/506	—	—	506/506	—	—
**Antihypercholesterolaemia drugs**	506/506	0.90 (0.74–1.10)	0.322	506/506	0.89 (0.73–1.09)	0.255
Statins	506/506	0.89 (0.72–1.10)	0.277	506/506	0.87 (0.70–1.07)	0.193
Cholesterol absorption inhibitors	506/506	0.98 (0.66–1.47)	0.939	506/506	1.00 (0.67–1.51)	0.984
Bile acid sequestrants	506/506	6.26 (0.87–45.06)	0.068	506/506	7.53 (1.03–55.26)	0.047
Nicotinic nacid derivatives	506/506	—	—	506/506	—	—
Fibrates	506/506	1.14 (0.28–4.58)	0.852	506/506	1.06 (0.26–4.28)	0.936
**Antidiabetes drugs**	506/506	0.57 (0.35–0.91)	0.018	506/506	0.59 (0.36–0.94)	0.028
Stimulation of beta cells	506/506	1.13 (0.47–2.74)	0.778	506/506	1.26 (0.52–3.07)	0.606
Alpha glucosidase inhibition	506/506	—	—	506/506	—	—
Alpha amylase inhibition	506/506	—	—	506/506	—	—
SGLT2 inhibition	506/506	—	—	506/506	—	—
Metformin	506/506	0.69 (0.40–1.17)	0.165	506/506	0.70 (0.41–1.19)	0.184
TZD	506/506	1.03 (0.15–7.36)	0.974	506/506	0.95 (0.13–6.80)	0.960
Insulin	506/506	0.44 (0.21–0.94)	0.034	506/506	0.44 (0.21–0.93)	0.033
**Anticonstipation drugs**	506/506	1.28 (0.81–2.00)	0.288	506/506	1.21 (0.77–1.90)	0.411
Stimulant laxatives	506/506	0.89 (0.40–2.00)	0.784	506/506	0.80 (0.35–1.80)	0.585
Osmotic laxatives	506/506	1.31 (0.33–5.26)	0.704	506/506	1.48 (0.36–6.01)	0.587
Bulk forming laxatives	506/506	3.49 (1.30–9.38)	0.013	506/506	3.63 (1.34–9.80)	0.011
Lubricant laxatives	506/506	—	—	506/506	—	—
Prokinetics	506/506	—	—	506/506	—	—
**Antidepression drugs**	506/506	0.89 (0.66–1.19)	0.424	506/506	0.90 (0.67–1.22)	0.510
SSRI	506/506	0.89 (0.60–1.31)	0.552	506/506	0.92 (0.62–1.36)	0.676
TCA	506/506	0.71 (0.44–1.15)	0.164	506/506	0.69 (0.43–1.12)	0.138
MAOI	506/506	—	—	506/506	—	—
Other antidepressants	506/506	2.78 (1.32–5.89)	0.007	506/506	3.10 (1.46–6.59)	0.003
**Antianxiety drugs**	506/506	0.89 (0.64–1.22)	0.461	506/506	0.86 (0.62–1.19)	0.364
Benzodiazepines	506/506	0.43 (0.11–1.74)	0.238	506/506	0.43 (0.10–1.77)	0.244
Barbiturates	506/506	—	—	506/506	—	—
Other anxiolytics	506/506	—	—	506/506	—	—

*Note:* The Cox regression analysis was adjusted for sex, age, qualifications, ethnicity, TDI, and BMI. Bold formatting has been applied exclusively to medication categories to denote classifications.

Abbreviations: ACE, angiotensin‐converting enzyme; ALS, amyotrophic lateral sclerosis; ARB, angiotensin II receptor blocker; BMI, body mass index; HR, hazard ratio; MAOI, monoamine oxidase inhibitor; SGLT2, sodium‐glucose cotransporter‐2; SSRI, selective serotonin reuptake inhibitor; TCA, tricyclic antidepressant; TDI, Townsend Deprivation Index; TDZ, thiazolidinedione.

## 4 Discussion

Using data from over 500 000 UK Biobank participants, our study compared ALS during its pre‐diagnostic phase. We observed an ALS incidence of 10.58 per 100 000 person‐years and a marked male predominance (male‐to‐female ratio of 2.9); early‐onset ALS and female gender may confer a survival advantage. Additionally, calcium channel blocker use may be positively associated with ALS. Pre‐diagnostically, falls were more common in ALS than in ad but less frequent than in PD, while psychiatric symptoms and autonomic dysfunction were less pronounced compared with PD.

On the one hand, our study supplements the existing evidence. Globally, a previous systematic review reported ALS incidence rates ranging from 0.26 per 100 000 person‐years in Ecuador to 23.46 per 100 000 person‐years in Japan [19]. While our estimated incidence of 10.58 per 100 000 person‐years falls within this range, it is still relatively high, which may be attributed to the older age distribution and the multiethnic background of this cohort [20]. Additionally, while neuropsychological impairments are not included in the diagnostic criteria for ALS, it is noteworthy that ALS patients may also experience such cognitive and psychological challenges. These may encompass a decline in executive memory functions, a marked increase in apathy and irritability, a noticeable neglect of personal hygiene, alterations in dietary habits, and the emergence of psychological issues such as depression, anxiety, and disrupted sleep patterns [21, 22]. Although these symptoms have the potential to manifest in the early phases of ALS, they are more frequently observed and tend to be more pronounced during the more advanced stages of the disease [23]. Some studies suggest that these psychiatric abnormalities may play a role in the progression of motor deficits and the prognosis of ALS [24]. This aligns with our finding that the use of antidepressants is associated with an increased risk of mortality following an ALS diagnosis. Besides, memory loss in ALS patients was already higher than in normal controls before diagnosis. Considering that approximately 15% of ALS patients meet the diagnostic criteria for frontotemporal dementia [25], as reported in the literature, this suggests a shared aetiology between ALS and other neurodegenerative diseases. However, the specific factors that lead to the distinct progression and differentiation of ALS from other degenerative diseases remain unknown.

On the other hand, this study provides new evidence, particularly in one aspect: exploring the preclinical characteristics of ALS. In the cross‐disorder analysis of symptoms in ALS, PD, and ad, we found that ALS shares many symptoms common to other neurodegenerative diseases, though it lacks distinct, specific manifestations. The most frequent pre‐diagnostic symptom in ALS, compared with ad, was falls; however, falls, psychiatric symptoms, and autonomic dysfunction in ALS were less prominent than in PD. Consequently, general physicians and even specialist neurologists might not initially recognize a diagnosis of amyotrophic lateral sclerosis due to the overlap in disease presentation with other conditions [1]. Additionally, we found that the use of calcium channel blockers prior to diagnosis may be associated with an increased risk of ALS. Calcium ions play a crucial role in various physiological processes, including muscle contraction, nerve signal transmission, blood clotting, and the regulation of enzyme activity [26]. Calcium channel blockers can influence the nervous system by reducing calcium influx into neurons, which may help regulate neurotransmitter release, reduce neuronal excitability, and protect against neurodegeneration [27]. Calcium channel blockers have been studied for their potential neuroprotective effects in conditions like ad and PD [28, 29]. However, our results suggest that calcium channel blockers may be harmful in ALS. Previous studies have indicated that hypertension and antihypertensive drug use are associated with a reduced risk of ALS [30, 31]. This appears to be a contradictory finding, as hypertension and antihypertensive drugs are generally considered to have opposing effects, yet both seem to offer a protective role for ALS. Considering that some studies suggest hypertension may increase ALS risk [32], current population‐based evidence remains conflicting and inconclusive. In animal research, previous studies have observed downregulation of Ca2 + in skeletal muscle mitochondria in ALS mouse models [33] and hyperactive intracellular calcium signaling [34]. However, these studies primarily focus on skeletal muscle and lack exploration of the key pathological site in ALS—the motor neurons. Future animal and clinical studies should focus more on hypertension and antihypertensive drugs to explore their potential impact on ALS.

Our study has limitations. First, ALS diagnoses in the UK Biobank are based on hospital records, death registers, and self‐reports, which may introduce delays, biases, and a skew towards more severe cases. Given the rarity of ALS, managing data for over 500 000 individuals to capture in detail about 700 ALS cases is challenging. Therefore, in the post‐diagnosis ALS analysis, we included only hospital data to ensure greater accuracy. Second, in our cross‐disease comparison of symptom profiles, the differences in the TDI among ALS, ad, and PD within the UK Biobank cohort were relatively small (standardized mean differences before matching < 0.1). This suggests a degree of regional homogeneity within the UK Biobank cohort, which is limited to participants from England, Wales, and Scotland. Therefore, future research using multiregional or international cohorts is needed to further validate our findings. Besides, the UK Biobank does not include genotype and phenotype data, making it difficult to distinguish between familial and sporadic ALS. Third, although the UK Biobank cohort included multiple ethnicities, its predominantly white composition limits the generalizability of our findings. Race is an important factor influencing ALS, as different ethnic groups may have distinct genetic backgrounds. For example, the expansion of the GGGGCC repeat in the C9ORF72 gene, a leading genetic cause of familial ALS, is more commonly found in individuals of European descent, particularly Southern Europeans like Italians and Spaniards [35]. Mutations in the Superoxide Dismutase 1 (SOD1) gene, which encodes superoxide dismutase 1, are also a well‐known cause of familial ALS and are most prevalent in Caucasian populations [36]. Additionally, variations in the Valosin‐containing protein and Optineurin genes have been identified as risk factors for ALS in African populations [37]. Therefore, the lack of race and genotype‐based stratification limits the depth of ALS research. Future studies should incorporate primary care and genetic data for precise ALS diagnosis, differentiate familial from sporadic cases, and expand ethnically diverse cohorts to enhance generalizability.

## 5 Conclusion

Prediagnostic presentations of falls are more frequent in ALS than in ad, but less frequent than in PD. However, ALS exhibits fewer psychiatric symptoms and autonomic dysfunction compared with PD. The use of calcium channel blockers may be associated with an increased risk of developing ALS in the future.

## Author Contributions

Chunyang Pang and Wen Cao contributed to data statistics and writing papers. Jiali Xie, Yaojia Li, Luyi Zhu, and Huan Yu participated in the data organization. Dongsheng Fan and Binbin Deng provided resources and designed the study.

## Ethics Statement

UK—the North West Multi‐centre Research Ethics Committee approved the Biobank (Ref: 11/NW/0382), with all participants providing written informed consent to participate in the UK Biobank study. This research was conducted using the UK Biobank resource under Application Number 108832.

## Conflicts of Interest

The authors declare no conflicts of interest.

## Supporting information


**Figure S1** Love plot showing the covariate balance before and after matching using nearest‐neighbour matching
**Figure S2.** Kaplan–Meier survival curves for 506 patients with amyotrophic lateral sclerosis since diagnosis from hospital
**Table S1.** Read codes and ICD‐10 codes used to identify health conditions
**Table S2.** ATC codes to identify treatment
**Table S3.** Medication classification based on ATC Codes
**Table S4.** Baseline characteristics of participants with and without ALS
**Table S5.** Covariate balance before and after matching across four models
**Table S6.** Prevalence of at least one occurrence during life course in ALS cohorts stratified by onset, survival, and sex.

## Data Availability

The data supporting the findings of this study are available on the UK Biobank project site and are subject to successful registration and application processes. Further details are available at https://www.ukbiobank.ac.uk/. Our research data are available upon reasonable request. The R code for the statistical analysis of this study is available upon request from Professor Dongsheng Fan and Professor Binbin Deng.
