# Supplementary material for: Prediagnosis Insights Into Amyotrophic Lateral Sclerosis: Clinical Symptoms and Medication Use
Source: J Cachexia Sarcopenia Muscle. 2025 Jul 11;16(4):e70003. doi: 10.1002/jcsm.70003 (PMC12246794; doi:10.1002/jcsm.70003)
Supplement: Supplementary file 1 — Figure S1 Love plot showing the covariate balance before and after matching using nearest‐neighbour matching Figure S2. Kaplan–Meier survival curves for 506 patients with amyotrophic lateral sclerosis since diagnosis from hospital Table S1. Read codes and ICD‐10 codes used to identify health conditions Table S2. ATC codes to identify treatment Table S3. Medication classification based on ATC Codes Table S4. Baseline characteristics of participants with and without ALS Table S5. Covariate balance before and after matching across four models Table S6. Prevalence of at least one occurrence during life course in ALS cohorts stratified by onset, survival, and sex. [file JCSM-16-e70003-s001.pdf]

Supplementary Material Catalogue

Supplementary Figure 1. Love plot showing the covariate balance before and after matching using nearest-neighbor matching. --2

Supplementary Figure 2. Kaplan-Meier survival curves for 506 patients with amyotrophic lateral sclerosis since diagnosis from hospital. ---3

Supplementary Table 1. Read codes and ICD-10 codes used to identify health conditions--------------4

Supplementary Table 2. ATC codes to identify treatment--5

Supplementary Table 3. Medication classification based on ATC Codes------------------------------------6

Supplementary Table 4. Baseline characteristics of participants with and without ALS ------------------7

Supplementary Table 5. Covariate balance before and after matching across four models---------------8

Supplementary Table 6. Prevalence of at least one occurrence during life course in ALS cohorts stratified by onset, survival, and sex---9

Supplementary figure 1. Love plot showing the covariate balance before and after matching using nearest-neighbor matching.

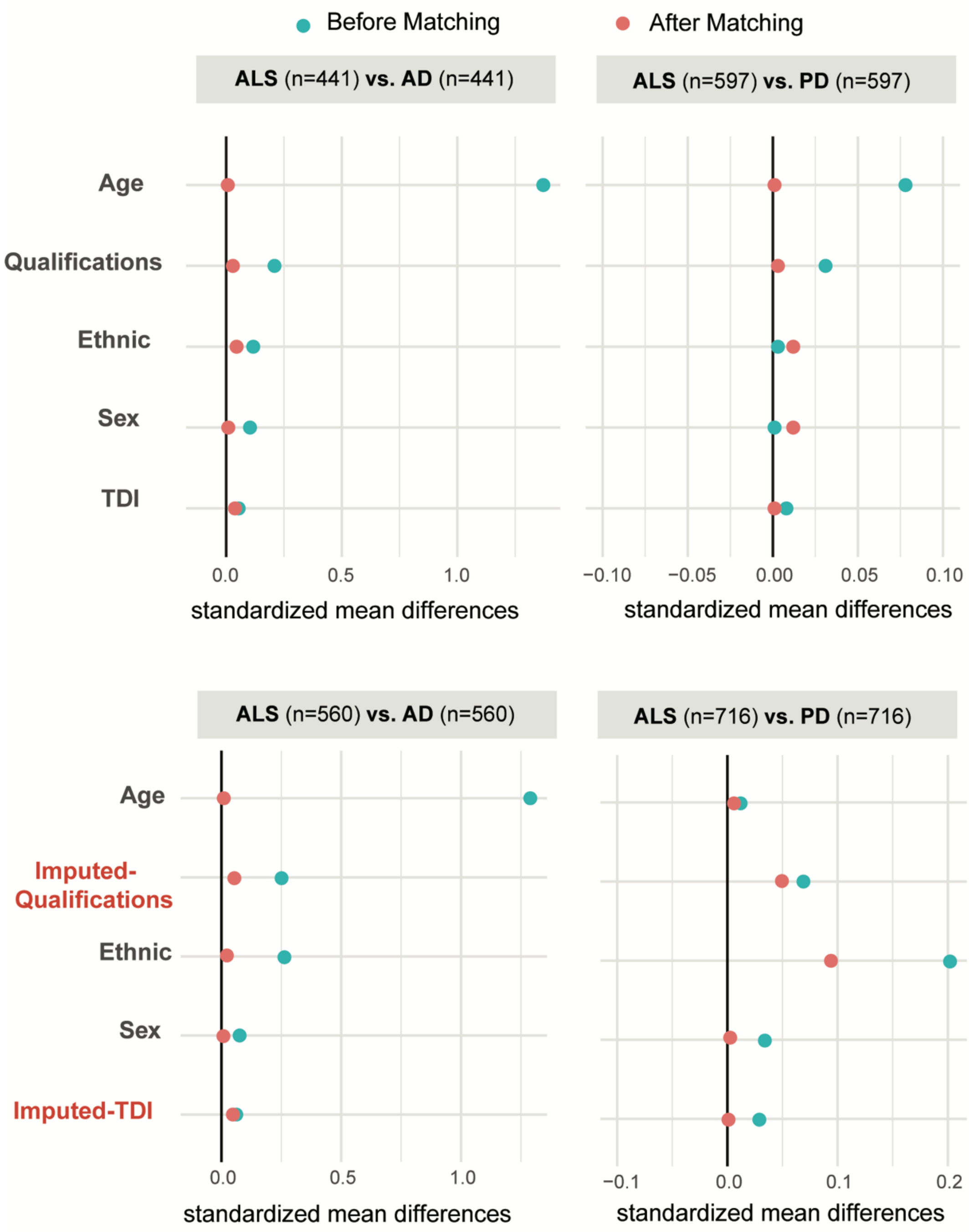

Abbreviations: ALS, Amyotrophic Lateral Sclerosis; AD, Alzheimer's Disease; PD, Parkinson's Disease; Cem, Coarsened Exact Matching; SMD, Standardized Mean Differences; TDI, Townsend Deprivation Index.

Supplementary Figure 2. Kaplan-Meier survival curves for 506 patients with amyotrophic lateral sclerosis since diagnosis from hospital.

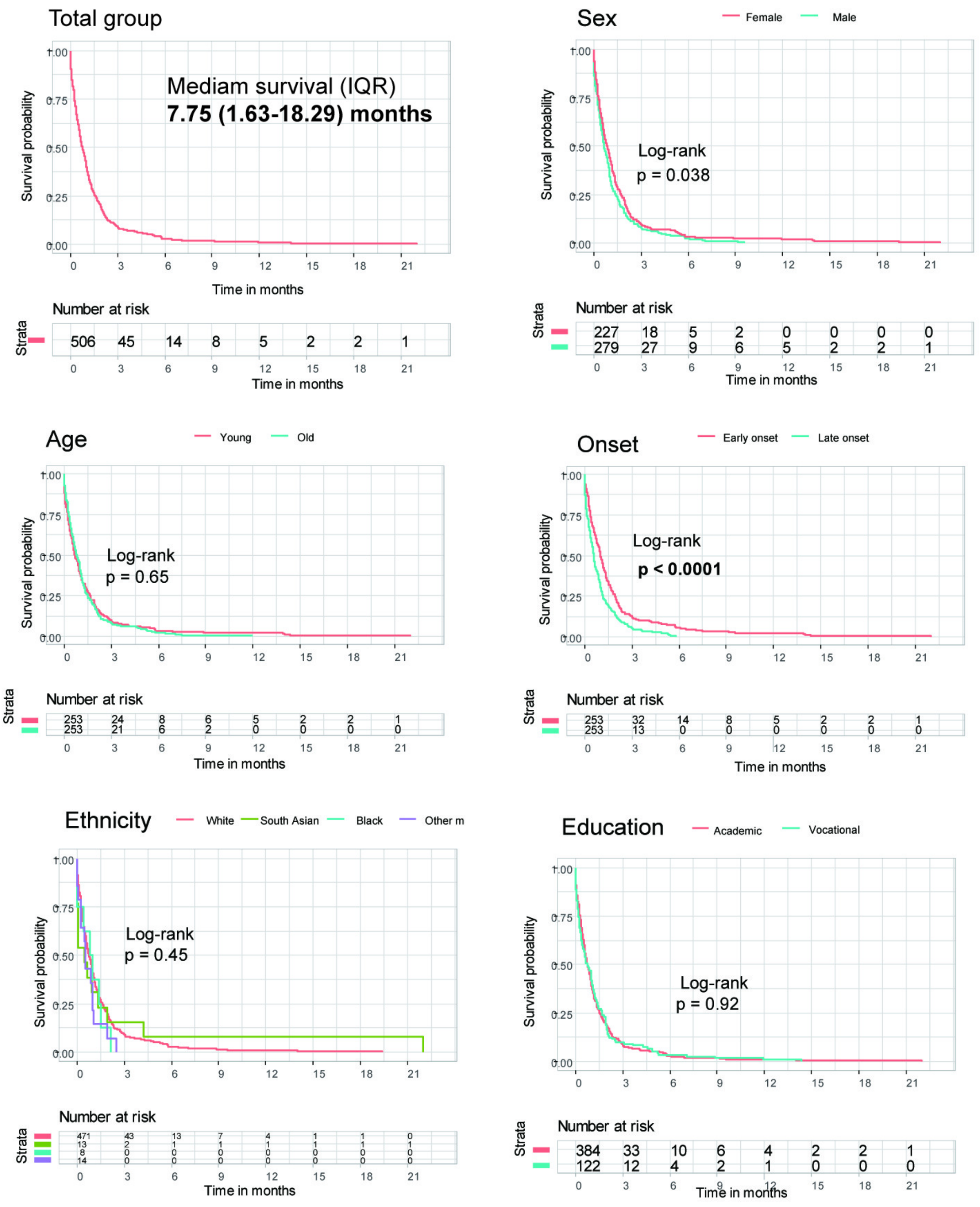

Supplementary Table 1. Read codes and ICD-10 codes used to identify health conditions.

Condition	Field ID	Read codes	Description
Constipation	41270	K59.0	Constipation
	20002	constipation	Constipation
Urinary dysfunction	41270	R35.0	Noninfectious urinary frequency
	41270	N39.0	Urinary tract infection, site not specified
	41270	N39.4	Other specified urinary incontinence
	41270	N32.81	Overactive bladder
	41270	N36.91	Functional disorder of male urethra, unspecified
	20002	Urinary frequency	Urinary frequency
Memoryloss	41270	R41.0	Disorientation
	41270	R41.1	Anterograde amnesia
	41270	R41.3	Other amnesia
Abnormal weight loss	41270	R63.0	Anorexia
	41270	R62.51	Failure to thrive in infant
	41270	R63.4	Abnormal weight loss
	41270	R64.4	Cachexia
	41270	F50.1	Atypical anorexia nervosa
	41270	F50.8	Other specified eating disorders
	41270	F50.9	Eating disorder, unspecified
	20002	Anorexia	Anorexia
Fall	41270	W00	Fall due to ice and snow
	41270	W01	Fall on same level from slipping, tripping and stumbling
	41270	W03	Other fall on same level due to collision with, or pushing by, another person
	41270	W05	Fall involving wheelchair
	41270	R29.6	Fall involving wheelchair
	41270	Z91.81	History of falling
	2296	Only one fall/more than one fall	Fall
Anxiety	130904	!.is.NA	Anxiety
	130906	!.is.NA	Anxiety
depression	130894	!.is.NA	Depression
	130896	!.is.NA	Depression
Sleep disorder	130920	!.is.NA	Sleep disorder
	131060	!.is.NA	Sleep disorder
Hypotension	131416	!.is.NA	Hypotension

Supplementary Table 2. ATC codes to identify treatment.

ATC code	Treatments	Family of treatments
CO3	Diuretics	Drugs used against hypertension
CO7	Beta-blocking agents	Drugs used against hypertension
CO8	Calcium-channel blockers	Drugs used against hypertension
CO9	Agent acting on angiotensin system	Drugs used against hypertension
C10AA	Statins	Drugs used against hypercholesterolemia
C10AB	Fibrates	Drugs used against hypercholesterolemia
C10AX	Other lipid modifying agents	Drugs used against hypercholesterolemia
A10A	Insulins and analogues	Drugs used against diabetes
A10BA	Biguanides	Drugs used against diabetes
A1OBB	Sulfonylureas	Drugs used against diabetes
A10BH	DDP4 inhibitors	Drugs used against diabetes
A10BJ	GLP-1 inhibitors	Drugs used against diabetes
A06	Laxatives	Drugs used against constipation
NO6A	Serotonin reuptake inhibitors	Drugs used against depression
NO5B	Benzodiazepines	Drugs used against anxiety

ATC, the Anatomical Therapeutic Chemical.

Supplementary Table 3: Medication classification based on ATC Codes.

Drug Category	Sub-category	ATC Code(s)
**Anti-diabetic Drugs**	Stimulation of beta cells	A10BB, A10BX, A10BJ, A10BH
	Alpha-glucosidase inhibition	A10BF
	Alpha-amylase inhibition	A10BF
	SGLT2 inhibition	A10BK
	Metformin	A10BA
	TZD	A10BG
	Insulin	A10A
**Anti-hypertensive Drugs**	ACE inhibitors	C09A, C09B
	ARB	C09C, C09D
	Beta blockers	C07A, C07B, C07C, C07D
	Calcium channel blockers	C08C, C08D
	Diuretics	C03A, C03B, C03C, C03D
	Alpha blockers	C02A, C02B
	Vasodilators	C02D
	Renin inhibitors	C09XA
**Anti-hypercholesterolemia drugs**	Statins	C10AA
	Cholesterol absorption inhibitors	C10AX
	Bile acid sequestrants	C10AC
	Nicotinic acid derivatives	C10AD
	Fibrates	C10AB
**Anti-depressants**	SSRI	N06AB
	TCA	N06AA
	MAOI	N06AF, N06AG
	Other antidepressants	N06AX
**Anti-anxiety drugs**	Benzodiazepines	N05BA
	Barbiturates	N05CA
	Other anxiolytics	N05BX
**Anti-constipation drugs**	Stimulant laxatives	A06AB
	Osmotic laxatives	A06AD
	Bulk forming laxatives	A06AC
	Lubricant laxatives	A06AA
	Prokinetics	A06AX

Supplementary Table 4. Baseline characteristics of participants with and without ALS.

	Non-ALS	ALS	P value
n	55292	753	
Basic information			
Sex, female, n (%)	28501 (51.5)	425 (56.4)	0.008
Ethnicity, n (%)			0.133
White	51388 (92.9)	707 (93.9)	
Mixed	378 (0.7)	2 (0.3)	
Asian or Asian British	1200 (2.2)	10 (1.3)	
Black or Black British	1010 (1.8)	20 (2.7)	
Other ethnic group	1316 (2.4)	14 (1.9	
Qualifications, n (%)			<0.001
Academic	41519 (83.8)	496 (77.8)	
Vocational	7978 (16.2)	142 (22.2)	
Age at admission, mean (SD)	53.24 (7.67)	56.26 (8.18)	<0.001
Health conditions during life course, n (%)			
Depression	6909 (12.5)	143 (19.0)	<0.001
Anxiety	4940 (8.9)	94 (12.5)	0.001
Sleep disorders	2331 (4.2)	78 (10.4)	<0.001
Memory loss	0 (0.0)	8 (1.1)	<0.001
Abnormal weight loss	45 (0.1)	18 (2.4)	<0.001
Constipation	84 (0.2)	23 (3.1)	<0.001
Urinary dysfunction	921 (1.7)	37 (4.9)	<0.001
Fall	11309 (20.5)	241 (32.0)	<0.001
Hypotension	2544 (4.6)	73 (9.7)	<0.001
Medications during life course, n (%)			
Drug against hypertension	16044 (29.0)	249 (33.1)	0.017
Drug against hypercholesterolemia	12146 (22.0)	197 (26.2)	0.007
Drug against constipation	2332 (4.2)	42 (5.6)	0.08
Drug against anxiety	3921 (7.1)	58 (7.7)	0.564
Drugs against depression	4433 (8.0)	86 (11.4)	0.001
Drug against diabetes	2130 (3.9)	27 (3.6)	0.778
Drugs against psychosis	24 (0.0)	1 (0.1)	0.776

#Other including Chinese, other ethnic group and those who do not know or prefer not to answer.

Academic qualification including College or University degree /A levels/AS levels or equivalent/O levels/GCSEs or equivalent; Vocational qualification including CSEs or equivalent/NVQ or HND or HNC or equivalent/Other professional qualifications.

Supplementary Table 5. Covariate balance before and after matching across four models.

Cohort	Index	Before matching		Nearest		Exact		Cem		Optimal	
		Case/ref.	SMD	Case/ref.	SMD	Case/ref.	SMD	Case/ref.	SMD	Case/ref.	SMD
**ALS** vs. **AD**	Age	636/2707	1.372	441/441	0.007	12/12	<0.001	207/396	0.427	636/636	0.543
	Qualifications	636/2707	0.103	441/441	0.009	12/12	<0.001	207/396	0.085	636/636	<0.001
	Ethnic	636/2707	0.209	441/441	0.029	12/12	<0.001	207/396	0.007	636/636	0.056
	Sex	636/2707	0.117	441/441	0.045	12/12	<0.001	207/396	0.204	636/636	0.102
	TDI	636/2707	0.054	441/441	0.038	12/12	<0.001	207/396	0.19	636/636	0.051
**ALS** vs. **PD**	Age	636/3563	0.008	597/597	0.001	32/32	0.042	330/534	0.068	636/636	0.056
	Qualifications	636/3563	0.031	597/597	0.003	32/32	<0.001	330/534	0.006	636/636	0.003
	Ethnic	636/3563	0.003	597/597	0.012	32/32	0.108	330/534	0.105	636/636	0.016
	Sex	636/3563	0.078	597/597	<0.001	32/32	<0.001	330/534	0.055	636/636	<0.001
	TDI	636/3563	0.001	597/597	0.012	32/32	<0.001	330/534	0.132	636/636	0.001
**ALS** vs. **AD**	Age	753/4445	1.289	560/560	0.010	19/20	0.047	297/729	0.455	753/753	0.412
	Qualifications#	753/4445	0.251	560/560	0.054	19/20	0.073	297/729	0.178	753/753	0.065
	Ethnic	753/4445	0.263	560/560	0.016	19/20	<0.001	297/729	0.108	753/753	0.119
	Sex	753/4445	0.076	560/560	0.004	19/20	0.038	297/729	0.031	753/753	0.067
	TDI#	753/4445	0.062	560/560	0.048	19/20	0.026	297/729	0.213	753/753	0.005
**ALS** vs. **PD**	Age	753/4809	0.012	716/716	0.006	34/35	0.103	407/744	0.091	753/753	0.004
	Qualifications#	753/4809	0.069	716/716	0.050	34/35	0.353	407/744	0.182	753/753	0.063
	Ethnic	753/4809	0.202	716/716	0.097	34/35	<0.001	407/744	0.167	753/753	0.150
	Sex	753/4809	0.034	716/716	0.003	34/35	0.089	407/744	0.038	753/753	0.051
	TDI#	753/4809	0.029	716/716	0.001	34/35	0.351	407/744	0.111	753/753	0.018

Abbreviations: ALS, Amyotrophic Lateral Sclerosis; AD, Alzheimer's Disease; PD, Parkinson's Disease; Cem, Coarsened Exact Matching; SMD, Standardized Mean Differences; TDI, Townsend Deprivation Index.

#Missing data imputed.

Supplementary Table 6. Prevalence of at least one occurrence during life course in ALS cohorts stratified by onset, survival, and sex.

Onset	Early	Late	P value
n	377	376	
Health conditions during life course, n (%)			
Depression	83 (22.0)	60 (16.0)	0.043
Anxiety	51 (13.5)	43 (11.4)	0.448
Sleep disorders	47 (12.5)	31 (8.2)	0.075
Memory loss	3 (0.8)	5 (1.3)	0.719
Abnormal weight loss	8 (2.1)	10 (2.7)	0.807
Constipation	14 (3.7)	9 (2.4)	0.401
Urinary dysfunction	18 (4.8)	19 (5.1)	0.993
Fall	134 (35.5)	107 (28.5)	0.045
Hypotension	35 (9.3)	38 (10.1)	0.796
ALS survival	Short survival	Long survival	P value
n	288	288	
Health conditions during life course, n (%)			
Depression	51 (17.7)	49 (17.0)	0.912
Anxiety	45 (15.6)	26 (9.0)	0.023
Sleep disorders	20 (6.9)	33 (11.5)	0.084
Memory loss	2 (0.7)	3 (1.0)	1
Abnormal weight loss	6 (2.1)	10 (3.5)	0.447
Constipation	6 (2.1)	10 (3.5)	0.447
Urinary dysfunction	12 (4.2)	16 (5.6)	0.561
Fall	85 (29.5)	89 (30.9)	0.785
Hypotension	29 (10.1)	25 (8.7)	0.668
Sex	Female	Male	P value
n	328	425	
Health conditions during life course, n (%)			
Depression	57 (17.4)	86 (20.2)	0.369
Anxiety	42 (12.8)	52 (12.2)	0.902
Sleep disorders	30 (9.1)	48 (11.3)	0.402
Memory loss	5 (1.5)	3 (0.7)	0.467
Abnormal weight loss	4 (1.2)	14 (3.3)	0.108
Constipation	6 (1.8)	17 (4.0)	0.133
Urinary dysfunction	18 (5.5)	19 (4.5)	0.638
Fall	113 (34.5)	128 (30.1)	0.236
Hypotension	30 (9.1)	43 (10.1)	0.747
